# Photoimmunotherapy of residual disease after incomplete surgical resection in head and neck cancer models

**DOI:** 10.1002/cam4.752

**Published:** 2016-05-11

**Authors:** Lindsay S. Moore, Esther de Boer, Jason M. Warram, Matthew D. Tucker, William R. Carroll, Melissa L. Korb, Margaret S. Brandwein‐Gensler, Gooitzen M. van Dam, Eben L. Rosenthal

**Affiliations:** ^1^ Department of Otolaryngology‐Head and Neck SurgeryUniversity of Alabama at BirminghamBirminghamAlabama; ^2^ Department of SurgeryUniversity Medical Center Groningen University of GroningenGroningenThe Netherlands; ^3^ Department of SurgeryUniversity of Alabama at BirminghamBirminghamAlabama; ^4^ Department of PathologyUniversity at BuffaloBuffaloNew York; ^5^ Department of Otolaryngology‐Head and Neck SurgeryStanford UniversityStanfordCalifornia

**Keywords:** Head and neck squamous cell carcinoma, IRDye700DX, panitumumab, photoimmunotherapy

## Abstract

Antibody‐based photodynamic therapy, or photoimmunotherapy (PIT), is a novel, targeted cancer therapy, which can serve as both a diagnostic and a therapeutic agent. The primary objective of this study was to evaluate the capacity of panitumumab‐IRDye700DX (Pan‐IR700) to eliminate microscopic tumor remnants in the postsurgical setting, which was accomplished using novel in vitro and in vivo models of residual disease after incomplete resection. Additionally, PIT was evaluated in fresh human‐derived cancer tissue. To determine a threshold for cellular regrowth after PIT, an in vitro assay was performed using a range of cells representing microscopic disease quantities. Long‐term growth inhibition was induced after treatment of 5 × 10^3^ and 1 × 10^4^ cells at 6 J. A novel in vivo mouse model of subtotal tumor resection was used to assess the effectiveness of Pan‐IR700 mediated PIT to eliminate residual disease and inhibit recurrence in the post‐surgical wound bed. Mice receiving surgical treatment plus adjuvant PIT showed a threefold and fourfold reduction in tumor regrowth at 30 days post PIT in the 50% and 90% subtotal resection groups, respectively (as measured by bioluminescence imaging), demonstrating a significant (*P* < 0.001) reduction in tumor regrowth. To determine the translatability of epidermal growth factor receptor (EGFR)‐targeted PIT, SCCHN human tissues (*n* = 12) were treated with Pan‐IR700. A significant reduction (*P* < 0.001) in ATP levels was observed after treatment with Pan‐IR700 and 100 J cm^−2^ (48% ± 5%) and 150 J cm^−2^ (49% ± 7%) when compared to baseline. Targeting EGFR with Pan‐IR700 has robust potential to provide a tumor‐specific mechanism for eliminating residual disease in the surgical setting, thereby increasing therapeutic efficacy, prolonging progression‐free survival, and decreasing morbidity.

## Introduction

Obtaining complete removal of tumor tissue while minimizing damage to surrounding healthy tissue with improved disease‐free and overall survival is the ultimate goal of surgical treatment of squamous cell carcinoma of the head and neck (SCCHN) 1, 2. Despite efforts to utilize more advanced surgical and medical technologies, the 5‐year survival rate has had modest improvement over the past three decades, remaining in the range of 50–55% 3, 4. Locoregional recurrence is the most common cause for treatment failure, and the prevalence of positive tumor margins is approximately 30% of surgical resections in current clinical practice 4, 5. Adjuvant treatments intended to eliminate residual disease after incomplete resections, including radiation and chemotherapy, can themselves fail to control disease recurrence and are associated with severe side effects. As such, there is an acute need for targeted treatment modalities that can facilitate total disease eradication to improve patient outcomes while limiting collateral damage of precious healthy tissues.

Antibody‐based photodynamic therapy, or photoimmunotherapy (PIT), is a novel, cancer‐targeted treatment modality that has demonstrated promise to improve the balance between efficacy and toxicity in the management of solid malignancies 6, 7, 8, 9, 10, 11. Traditional photodynamic therapy, while effective in killing cancer cells, employs nontargeted photosensitizers that induce light‐dependent cytotoxicity to noncancerous cells, resulting in severe side effects and limiting clinical translatability 7. Alternatively, PIT utilizes the specificity of antibody binding to deliver therapeutic phototoxicity to malignant cells aberrantly overexpressing target receptors while sparing adjacent normal tissues 7, 8, 9, 10.

**Figure 1:**
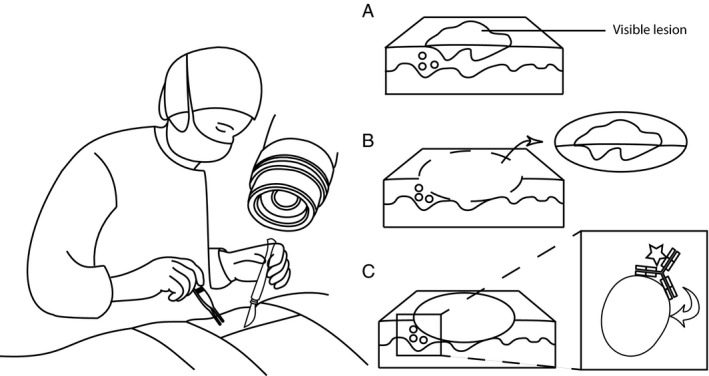
PIT‐guided surgery. The mAb‐photosensitizer construct is administered systemically. (A–B) The tumor‐targeted mAb will allow for real‐time fluorescent‐guided surgery, (C) but will also generate highly reactive singlet oxygen molecules, which directly kills unresectable microscopic residual disease.

However, the strategy of using antibodies to target delivery of an optically active molecule to cancer cells is not unique to PIT. The field of fluorescence‐guided surgery has demonstrated the ability of a number of various fluorophore‐antibody combinations to successfully provide cancer‐specific fluorescent contrast to help delineate cancer margins during surgical resection 12. Given the obvious overlap between these applications, experts in both fields have recognized the potential to combine the technologies to explore a dual diagnostic and therapeutic paradigm, and have already demonstrated early success in this proposed model 6, 7, 8, 9. In this approach, antibodies are conjugated to a fluorescent photosensitizer, such as IRDye700DX, and act as targeting vectors that specifically deliver the photosensitizer to the tumor. Upon antibody binding to cancer cells, a relatively brief exposure from an external light source can be used for fluorescence imaging to localize the tumor for diagnostic purposes (Fig. 1A and B), while high‐energy excitation from an external light source produces cytotoxic light emissions from the photosensitizer that induce localized cell death (Fig. 1C) 7, 8. In the intraoperative or endoscopic setting, this technique could be applied to the post‐resection wound bed as a surgical adjuvant to specifically treat unrecognized positive margins or microscopic residual disease.

While research has been conducted to assess the ability of fluorescent photosensitizers to provide tumor‐specific contrast (as in Fig. 1A) and to quantify tumor suppression in in vitro and whole tumor in vivo models 6, 7, 8, 9, 10, 11, there are no studies specifically exploring the feasibility or value of a fluorescent photosensitizer in the proposed role of treating residual disease in a post‐surgical wound bed (Fig. 1B). Therefore, the objective of this study was to evaluate the performance of the fluorescent photosensitizer IRDye700DX conjugated to the anti‐epidermal growth factor receptor (EGFR) monoclonal antibody panitumumab (panitumumab‐IRDye700DX; Pan‐IR700) as a PIT agent to eliminate microscopic residual disease in a wound bed following incomplete surgical resection in a model of SCCHN. This was accomplished using an in vitro model assessing a range of quantities representing microscopic disease to establish the cellular threshold of tumor suppression and a novel in vivo model of residual disease after incomplete resection.

Obviously, given the ability of Pan‐IR700 to induce tumor‐specific cell death without toxic side effects and its potential as a dual therapeutic and diagnostic agent, the feasibility and value of clinical translation of this technology into humans is quite promising. However, data to describe the therapeutic potential of PIT using IRDye700DX conjugated to monoclonal antibodies (mAb) is currently limited to a single laboratory 7, 9, 10, 11, and no studies have been conducted to test this agent in human tissue. Therefore, a second objective of this study was to explore the phototoxic efficacy of Pan‐IR700 in an ex vivo model using fresh, patient‐derived SCCHN tissue samples.

## Material and Methods

### Cell lines and animal models

For this study, the luciferase‐expressing SCCHN 1 (SCC‐1‐Luc) was used. The cell line was obtained from Dr. Thomas Carey (University of Michigan, Ann Arbor, MI) and maintained in Dulbecco Modified Eagle's Medium (DMEM) supplemented with 10% fetal bovine serum (FBS) and 1% L‐glutamine. The cell line was cultured at 37°C in a humid atmosphere with 5% CO_2_. Cells were passaged at 80–90% confluence and harvested with 0.05% trypsin. A hemocytometer with trypan dye exclusion was used to quantify cell numbers.

For the animal models, athymic female nude mice, aged 5–6 weeks (Frederick Cancer Research, Frederick, MD) were obtained and housed in accordance with guidelines and regulations of the Institutional Animal Care and Use Committee (IACUC). For subcutaneous inoculation, 2 × 10^6^ cells/100 *μ*L Dulbecco's modified Eagle medium (without FBS) were subcutaneously implanted in the left flank. Tumor growth was monitored by bioluminescence imaging (BLI, photons/sec/cm^2^/sr; IVIS‐100, Caliper Life Sciences, Waltham, MA) and visual inspection two times per week. Eight weeks post implantation, mice were sorted into four groups (*n* = 3) based on tumor luciferase expression as determined by BLI to achieve equal distribution of tumor size and growth rate. All animal experiments were performed according to approved IACUC protocols.

### Panitumumab‐IRDye700DX conjugation

Conjugation of the fully humanized anti‐EGFR mAb panitumumab to the photosensitizer IRDye700DX (absorption max: 689 nm, emission max: 700 nm) was performed as previously described 8. Briefly, panitumumab (Pan; Vectibix, Amgen, Thousand Oaks, CA; 177 kDa) was diluted to 5 mg mL^1^ in PBS. IRDye700DX NHS ester (IR700; LI‐COR Biosciences, Lincoln, NE; 2.0 kDa), which allows for reproducible, single‐step covalent protein conjugations 7, was conjugated to panitumumab for 2 h at room temperature in the dark, at a molar ratio of 2:1. After purification using a desalting spin column (Pierce Biotechnology, Rockford, IL) to remove unconjugated dye, the final protein concentration and number of dye molecules per protein were measured by UV‐Vis spectroscopy (Nanodrop 200‐c, ThermoScientific, Wilmington, DE).

### In vitro microscopic disease model proliferation assay

SCC‐1‐Luc cells were seeded into a 24‐well, black‐welled plates (Wallac) at a concentration of 5 × 10^3^, 1 × 10^4^, 2.5 × 10^4^, and 5 × 10^4^ cells per well. One day after seeding, cells were treated accordingly: (1) no treatment; (2) PIT at 6 J cm^−2^; (3) Pan‐IR700 and PIT at 6 J cm^−2^. Pan‐IR700 dose was 10 *μ*g mL^−1^. Cell viability was determined by BLI at 8 h, 1, 2, 4, and 5 days after PIT mediated or not mediated Pan‐IR700 treatment. BLI signal was measured daily, and the fold increase in BLI signal over time from the baseline value was calculated.

### In vivo xenograft model of residual disease after incomplete resection

The mice were sorted into four groups (*n* = 3): subtotal surgical resection of 90% or 50% + 300 *μ*g Pan‐IR700 + 100 J; subtotal surgical resection of 90% or 50% + 300 *μ*g Pan‐IR700 alone as previously described 13. Pan‐IR700 was dosed intravenously via tail vein. In mice in the 90% subtotal resection group, 10% of the tumor was left in the wound cavity; and in mice in the 50% subtotal resection group, 50% of the tumor was left in the wound cavity, as determined by weight. All mice underwent identical surgical procedures. Briefly, 1 day post Pan‐IR700 injection, mice received 2.5 mg D‐luciferin intraperitoneally (IP) 10 min prior to tumor resection. After anesthetizing the mice, the tumors were removed and appropriate amounts of tumor (i.e., 90% or 50%) was removed from the resected tumor, and remaining amounts (resp. 10% and 50%) placed back in the wound bed. BLI was then performed on the wound bed to confirm the presence of luciferase‐positive cancer. Fluorescence imaging of the wound bed was also performed using a closed‐field imaging system (Pearl, LI‐COR Biosciences, Lincoln, NE) specifically designed for the IR700 fluorescent spectrum. Following tumor imaging, dependent on the group, the wound bed was exposed to 100 J or 0 J. Subsequently, the wound bed was closed using 6‐O fast‐absorbing plain gut suture using a PC‐1 conventional cutting 3/8 circle needle (1916, Ethicon, San Angelo, TX). The mice were subcutaneously injected with a 100 *μ*g cocktail of 1 mg mL^−1^ carprofen and 20 *μ*g mL^−1^ buprenorphine to relieve residual pain from surgery. The mice were allowed to heal for 4 days before follow‐up BLI began. BLI signal was determined at each imaging time point, and fold increase in BLI signal at each time point from the baseline measurement immediately following tumor fragment reimplantation was calculated. All applicable institutional guidelines for the care and use of animals were followed.

### Ex vivo model of patient‐derived tissue samples

Tumor specimens (*n* = 12) were obtained from histologically confirmed SCCHN patients at the time of resection and placed in complete culture media (DMEM supplemented with 10% FBS, 1% penicillin‐streptomycin, and 1% gentamycin). Within 1 h post specimen retrieval, multiple tissue slices (3–6 replicate slices per treatment group) measuring approximately 4 mm in diameter and 1–2 mm in thickness were obtained via biopsy punches and sharp dissection. Tumor slices were weighed and randomly placed into individual wells of 24‐well plates in 0.5 mL of complete media supplemented with unlabeled panitumumab (0.02 mg mL^−1^), Pan‐IR700 (0.02 mg mL^−1^), or no antibody and incubated at 37°C in 5% CO_2_ for 24 h. Tumor slices were then washed three times in PBS. Complete media was added to the specimens and LED illumination was applied at 50 J cm^−2^, 100 J cm^−2^ , or 150 J cm^−2^ to individual antibody‐treated groups. Twenty‐four hours post PIT, an ATP viability assay was performed on sonicated tissue slices as previously described 14. Therapeutic effect is expressed as percent ATP level compared to the control. All patients were given informed consent and the Institutional Review Board approval was obtained for procurement and ex vivo treatment of tissue specimens.

### Flow cytometry

Patient‐derived tumor slices were incubated with Pan‐IR700 in a 1:10 serial dilution (20 *μ*g mL^−1^ – 0.02 *μ*g mL^−1^) in triplicate. After washing, wells were fluorescently imaged using a fluorescence scanner (Odyssey, LI‐COR). After imaging the tumor slices in the fluorescent scanner, specimens were homogenized to generate single‐cell suspensions that were subsequently analyzed for fluorescent count in the 700 channel using a flow cytometer (Accuri Cytometers inc, Ann Arbor, MI).

### Histology

Patient‐derived tumor slices were incubated with Pan‐IR700 at a concentration of 20 *μ*g mL^−1^ in triplicate. After washing, specimens were formalin‐fixed, paraffin‐embedded, cut and mounted on glass slides. IR700 fluorescence intensity was measured on specimen‐mounted slides using a fluorescence scanner (Odyssey, LI‐COR). Immunohistochemistry was performed to evaluate EGFR density (anti‐human EGFR Ab‐10, Thermoscientific, Waltham, MA). Subsequently, a board‐certified pathologist confirmed the presence of tumor within EGFR‐stained sections (MBG).

### Statistics

Data are expressed as means ± standard error of the mean (s.e.m.). Independent and paired samples t‐tests were used to compare treatment effects with that of controls. For all statistical analyses, SPSS (version 21.0) was used. *P*‐value <0.05 was considered to indicate a statistically significant difference.

## Results

### PIT induces growth inhibition of 10,000 cells in an in vitro mode of microscopic residual disease

**Figure 2:**
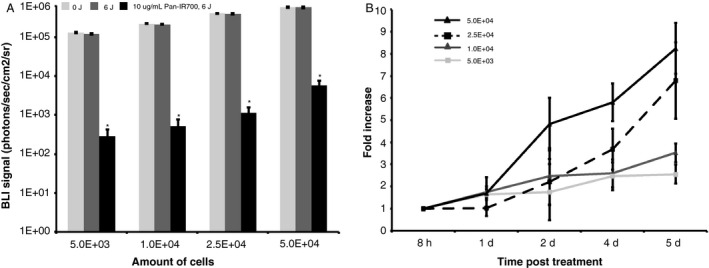
Pan‐IR700‐mediated photoimmunotherapy in vitro. (A) A significant reduction in viability was obtained with the treatment of 10 *μ*g/mL Pan‐IR700‐mediated PIT at 6 J compared to control cells exposed to 0 J and 6 J only (*P *< 0.001). (B) Proliferation assay after Pan‐IR700 (10 *μ*g/mL)‐mediated PIT at 6 J shows long‐term growth inhibition in 5 × 10^3^ and 1 × 10^4^ cells (*P *< 0.001). BLI, BioLuminescence Imaging (photons/sec/cm^2^/sr).

To establish a threshold for cellular regrowth after PIT, an in vitro assay was performed using luciferase‐positive SCC‐1‐Luc cells of varying amounts to represent microscopic disease quantities. Cells were treated with 10 *μ*g mL^−1^ Pan‐IR700 followed by 6 J of LED illumination. Significant cytotoxicity was achieved with treatment of 6 J and Pan‐IR700, but no cytotoxicity was observed with 6 J alone in any cell concentration (Fig. 2A). Longer term growth inhibition was seen in lower density cell platings treated with Pan‐IR700 mediated PIT (Fig. 2B; *P* < 0.05 at 4 days and 5 days post PIT). At day 5, a 47‐fold and 10‐fold increase in luminescent counts was observed for the 2.5 × 10^4^ and 5 × 10^4^ cells compared to the 5x10^3^ and 1x10^4^ cells, respectively. However, in the groups with 5 × 10^3^ and 1 × 10^4^ cells, long‐term growth inhibition was observed in response to Pan‐IR700 mediated PIT.

### PIT with Pan‐IR700 suppresses disease recurrence in an animal model of incomplete resection

**Figure 3:**
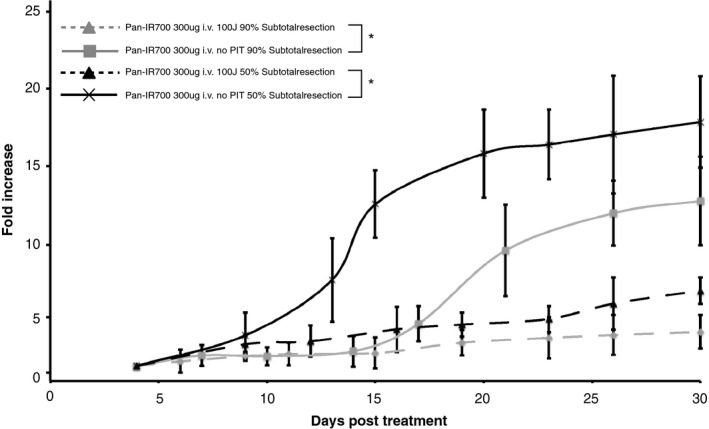
Bioluminescence imaging of tumor viability after surgical resection. Mice treated with PIT exhibit significantly less tumor regrowth in each group compared with mice exposed to Pan‐IR700 alone (*P *< 0.05).

To assess the efficacy of PIT using Pan‐IR700 as a postsurgical treatment option to eradicate residual disease, a novel in vivo model of incomplete resection was developed. Mice receiving surgical treatment plus adjuvant PIT on the surgical wound bed showed a significant (*P* < 0.001) reduction in growth over the 4‐week observation period compared to mice that did not receive adjuvant PIT (Fig. 3). No significant change in tumor size was observed over time in mice receiving surgical treatment plus adjuvant PIT (*P* < 0.05). However, in mice that received surgical therapy only, a significant increase in tumor size and BLI signal was observed in both subtotal resection groups from day 15 on (*P* < 0.05). As detailed in Figure 3, mice that underwent postoperative PIT on the wound bed showed a threefold and fourfold reduction in tumor regrowth at 30 days post PIT in the 50% and 90% subtotal resection groups respectively, as measured by BLI.

### PIT with Pan‐IR700 decreases cell viability in patient‐derived SCCHN tissue

**Figure 4:**
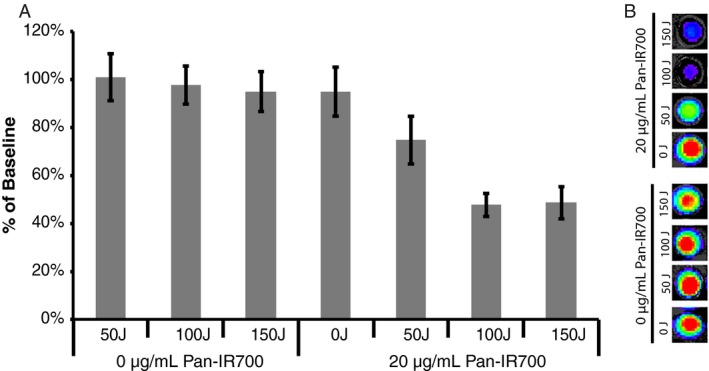
Pan‐IR700‐mediated PIT in SCCHN patient specimens. (A) Viability for head and neck cancer tissue slices (*n* = 12) per treatment group. A significant reduction in ATP levels was obtained in tissues treated with 20 *μ*g/mL Pan‐IR700 and 100 J and 150 J (*P *< 0.001). (B) Representative data are depicted for each treatment combination.

To determine the translatability of EGFR‐targeted PIT in humans, we obtained SCCHN human tissue samples (*n* = 12) and treated ex vivo with Pan‐IR700 (Fig. 4). Values are reported as mean percentage ATP levels of untreated controls (0 J cm^−2^, 0 *μ*g mL^−1^). Tissue specimens exposed to light alone (50 J cm^−2^, 100 J cm^−2^ or 150 J cm^−2^) did not demonstrate a significant difference from baseline (*P* > 0.05). Moreover, tissue treated with Pan‐IR700 alone did not demonstrate a significant difference from baseline in tissue viability (*P* > 0.05). A significant decrease in cell viability was not observed in tissue treated with Pan‐IR700 and 50 J cm^−2^. However, a significant reduction (*P* < 0.001) in ATP levels was observed after treatment with Pan‐IR700 and 100 J cm^−2^ (48% ± 5%) and 150 J cm^−2^ (49% ± 7%) when compared to baseline. There was no significant difference in cell viability levels between these two groups.

### Pan‐IR700 localizes to EGFR+ tumor regions in human‐derived SCCHN tumor specimens

**Figure 5:**
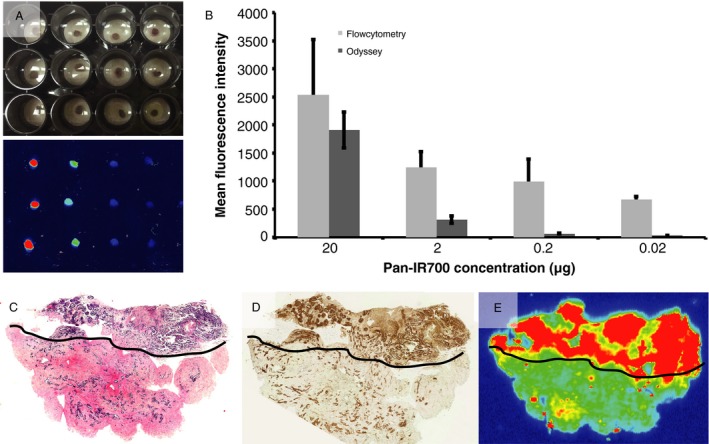
Histology. (A) Fluorescence was shown to stain the tissues in a dose‐dependent manner when imaged in wells with the fluorescent Odyssey scanner. (B) After homogenizing the tissue, dose‐dependent cellular fluorescence was shown using flow cytometry. Representative H&E, EGFR, and fluorescence image. (C**–**E) Tumor‐containing areas (annotated by black line) contained relatively higher levels of EGFR expression and highest fluorescence signal when imaged using the fluorescence Odyssey scanner.

Human SCCHN cells and tissues were treated with Pan‐IR700 and fluorescence histology was compared to histopathology to assess the specificity of Pan‐IR700 binding. Pan‐IR700 was shown to stain the tissue in a dose‐dependent manner and generated fluorescence when imaged in wells using a fluorescent flatbed scanner (Fig. 5A and B; Odyssey, LI‐COR). Fluorescence localization was explored in whole tissue specimens and in single‐cell suspensions. When tumor specimens were homogenized and cellular fluorescence was quantified using flow cytometry, a dose‐dependent expression of fluorescence was observed (Fig. 5B). When specimens were analyzed histologically, tumor‐containing areas, identified using H&E staining (Fig 5C; annotated by board‐certified pathologist [MBG]), expressed higher levels of EGFR compared to normal tissue (Fig 5D). The areas of highest fluorescence were shown to co‐localize with areas of high EGFR expression and cancer‐containing tissue (Fig. 5E; Odyssey, LI‐COR).

## Discussion

Surgical resection and adjuvant radiotherapy or chemoradiation is considered the gold standard for treatment of SCCHN 1, 2. However, recurrence rates remain high (50–55%), and predominantly occur locoregionally due to microscopic residual cancer cells left in the post‐resection wound bed 3, 4. The complex geometry and topography of head and neck anatomy, as well as the abundance of vital structures, make complete resections with disease‐free margins particularly challenging in this region. In addition to the severe side effects associated with adjuvant treatments, the devascularized and hypoxic nature of the post‐surgical wound bed compromises the delivery of any adjuvant systemic drugs and radiotherapy, respectively. Photoimmunotherapy is a promising treatment modality that could provide cancer‐targeted therapy to eliminate residual disease without the damage to normal tissues and side effects that current adjuvant treatments and even traditional photodynamic therapy entail 7, 8, 9, 10, 11.

Given the development of molecular dyes that can act as both fluorophores and photosensitizers, such as the IRDye700DX explored in this study, PIT has long been envisioned to function in the clinical setting as a valuable combination of the two technologies 7, 8, 9, 10, 11. Antibody‐based targeted delivery of photosensitizing fluorophores to cancer offers the potential for real‐time, intraoperative localization to guide resection with fluorescence and localized, intra‐ and post‐operative phototoxic treatment of any residual disease with a single agent. The application of this dual technique in the wound bed is particularly pertinent, as the relatively superficial location of residual cancer in the wound bed optimizes both technologies by eliminating artifacts like attenuation and scattering that can limit efficacy in deeper tissues 15. Furthermore, administration of the agent prior to wound bed devascularization, the long half‐life of antibody‐based probes, the mechanism of action, and the superficial and localized nature of PIT imply that this treatment would not be limited by the poorly vascularized and hypoxic environment of the postsurgical wound bed.

Current literature concerning PIT includes a number of studies exploring the fluorescence potential of photosensitizing fluorophores, like Pan‐IR700, as well as its phototoxic efficacy to treat whole tumors 7, 8, 9, 10, 11. However, there is a paucity of data actually exploring the efficacy of PIT in its proposed role as a postsurgical adjuvant treatment. Thus, the primary objective of this study was to elucidate the efficacy of PIT using Pan‐IR700 to eradicate microscopic residual disease in the wound bed after incomplete resection, which was assessed using in vitro and in vivo models of microscopic and residual disease, respectively. We also explored, for the first time, the performance of PIT in fresh human SCCHN tissue.

First, PIT was tested on a range of cells representing increasing microscopic quantities to establish a numerical threshold of effective phototoxic therapy. Long‐term growth inhibition after Pan‐IR700 mediated PIT treatment was observed for 5 × 10^3^ and 1 × 10^4^ cells in this in vitro model. However, regrowth eventually occurred in the 2.5 × 10^4^ and 5 × 10^4^ groups, suggesting that, for a single PIT treatment (6 J cm^−2^, 10 *μ*g mL^−1^ Pan‐IR700) the threshold of effective growth suppression lies somewhere between 10,000 and 25,000 cells. This finding suggests that PIT is more effective with fewer cells, further supporting its use to treat residual microscopic disease instead of large, unresected tumor masses.

Assuming that there are approximately 1 × 10^8^ cells in 1 cm^3^ (approximately 1.0 g) of tumor tissue 16, 10,000 cells would represent less than 1 mm^3^. Given that 1 cm^3^ is generally presumed to be the amount of tumor discernible by the human eye, there is likely a range of subclinical disease that cannot be effectively treated with a single PIT treatment at this time. However, repeated PIT treatments have demonstrated significantly improved cell killing compared to single‐ treatment regimens 11. If this in vitro study were presumed to be translatable in a biological system, one could conclude that multiple treatments would be recommended in the case of positive margins or suspected incomplete resections; however, given the noninvasive nature of light therapy and the data suggesting minimal side effects with PIT, this would arguably be a welcome alternative to chemo‐ or radiotherapy, although testing this model with repeated treatments would be needed to draw definitive conclusions. It is important to consider, however, that a single, intraoperative light treatment easily applied to the exposed wound bed of any patient with apparently “clean” margins not requiring adjuvant therapy would theoretically be substantial enough to effectively treat the ultra‐microscopic nests (well under 10,000 cells) that may not be included in frozen sections taken of a margin. Furthermore, some fluorescence imaging systems are reportedly able to detect as little as 0.1 mg of tumor (unpublished data), suggesting that using fluorescent photosensitizers in their dual diagnostic and therapeutic role could narrow the gap between tumor able to be visually resected and residual microscopic disease amenable to a single PIT treatment.

More importantly, long‐term growth inhibition of residual disease was observed in a novel mouse model of incomplete surgical resection, in which either 50% or 10% of the tumor was left in the wound bed. In order to ensure accuracy and establish standardization in this model, the entire tumor was resected and weighed to provide a quantitative method of determining the precise amount of tumor to be returned to the wound bed. In this study, a single NIR light irradiation was effective in inducing long‐term growth inhibition. Importantly, greater growth suppression was achieved in the 90% resection group, once again suggesting increasing efficacy of this treatment modality with decreasing amounts of residual tumor. Ultimately, however, local recurrences were seen in treated mice. Similar findings were also reported by Mitsuanga et al. in a xenograft mouse model using a single PIT treatment 7. A later publication by the same group demonstrated improved control of local tumor recurrence with repeated administration of PIT in treated mice with whole tumors 11, suggesting that multiple treatments would likely promote eradication of residual disease in an incomplete resection model. Furthermore, the pieces of tumor reimplanted in this model, even in the 90% resection group, were macroscopically visible, and therefore much larger than microscopic amounts left in a true post‐resection wound bed. Thus, it is reasonable to expect even greater control of locoregional tumor recurrence in a more realistic clinical setting.

The majority of studies describing the efficacy of PIT have been performed using in vitro and in vivo xenograft studies 7, 8, 9, 10, 11. The major disadvantage to this, of course, is the lack of clinical translatability of these highly preclinical models 14. The tumor and its surroundings may have a significant influence on the therapeutic potential of PIT, especially given that the mechanism of phototoxic cell death induced by Pan‐IR700 remains poorly understood 7. Therefore, for what we believe to be the first time, PIT‐induced cytotoxicity using Pan‐IR700 was assessed in patient‐derived tumor specimens. A significant reduction in cell viability was observed in the presence of the photosensitizer and 100 or 150 J cm^−1^ of PIT, further validating the feasibility and potential value of this technology to be advanced to clinical studies.

After human SCCHN tissue samples were incubated with Pan‐IR700, fluorescence histology demonstrated a strong correlation between fluorescence, EGFR expression, and cancer, and did so in a dose‐dependent manner. This study demonstrates highly specific binding of Pan‐IR700, which is consistent with testing of target‐specific binding of this agent using various assays by our laboratory and others using Pan‐IR700 7, 8, 9.

In conclusion, our results indicate that PIT using Pan‐IR700 demonstrates great potential as an adjuvant treatment modality to intraoperatively and postoperatively eliminate residual microscopic disease following incomplete resections, thereby aiding the surgeon in obtaining a more radical resection while preserving as much functionality as possible with minimal collateral damage to normal tissues. Furthermore, the efficacy of this technology on fresh human cancer tissue adds to the growing body of evidence supporting the clinical translation of this technology.

## Conflict of Interset

None declared.
